# Age of Language Acquisition Affects Topology of Brain White Matter Network: A Diffusion Tensor Magnetic Resonance Imaging Study

**DOI:** 10.3390/brainsci16080878

**Published:** 2026-08-18

**Authors:** Yibing Zhang, Liu Tu, Kei Omata, Guanying Chen, Xiaojin Liu, Tsunehiko Takamura, Lihan Zhang, Noritaka Wakasugi, Caiyan Zou, Hiroki Togo, Jinqiao Zhang, Zhongshi Li, Dongsong Li, Takashi Hanakawa

**Affiliations:** 1 College of Foreign Studies, Jinan University, Guangzhou 510632, China; katrina_zyb@163.com (Y.Z.); ingrid@stu2025.jnu.edu.cn (G.C.); 18270101899@163.com (L.Z.); 18173132919@163.com (C.Z.); zhongshili2025@163.com (Z.L.); tlids@jnu.edu.cn (D.L.); 2 Department of Advanced Neuroimaging, Integrative Brain Imaging Center, National Center of Neurology and Psychiatry, Tokyo 187-8551, Japan; kei-o@tc4.so-net.ne.jp (K.O.); t.taka.ivory@gmail.com (T.T.); n.wakasugi@ncnp.go.jp (N.W.); togo.hiroki.6a@kyoto-u.ac.jp (H.T.); hanakawa.takashi.2s@kyoto-u.ac.jp (T.H.); 3 Center for Educational Science and Technology, Beijing Normal University, Zhuhai 519087, China; xiaojin.liu@bnu.edu.cn; 4 Medical AI & Deep Learning Research Lab, Department of Strategic Research, National Center for Child Health and Development, Tokyo 157-8535, Japan; 5 Department of Behavioral Medicine, National Institute of Mental Health, National Center of Neurology and Psychiatry, Tokyo 187-8553, Japan; 6 Integrated Neuroanatomy and Neuroimaging, Kyoto University Graduate School of Medicine, Kyoto 606-8501, Japan; 7 College of Chinese Language and Culture, Jinan University, Guangzhou 510610, China; jjjqqqzzz@126.com

**Keywords:** age of language acquisition, tonal language, diffusion tensor imaging, white-matter brain network

## Abstract

**Highlights:**

**What are the main findings?**

- This study investigated the association between age of acquisition (AoA) in language and the topology of white matter structural networks in Japanese-Chinese/Chinese-Japanese speakers. Early speakers exhibited higher nodal clustering coefficient (NCp) in the right precuneus and higher betweenness centrality (BC) in the right Heschl’s gyrus (HG) than late bilinguals.
- The findings suggest that earlier L2 acquisition is associated with enhanced local network organization in the precuneus, a region involved in cognitive control. Earlier Chinese acquisition was associated with greater network centrality in the right HG, supporting its role in auditory and pitch-related language processing.

**What are the implications of the main findings?**

- The timing of language acquisition shapes the structural organization of white matter networks in specific brain regions.
- Earlier L2 acquisition may facilitate the development of neural networks supporting cognitive control, whereas earlier exposure to a tonal language such as Chinese may promote structural adaptations in auditory regions specialized for pitch processing.

**Abstract:**

Background/Objectives: In bilinguals, experience-based inter-individual differences, including language acquisition age, influence the functional and structural organization of the brain. Although many studies have explored the effects of age of acquisition on non-tonal languages, only a few studies have investigated the relationship between the age of tonal language acquisition and brain networks. Therefore, the aim of the study is to investigate how the age of language acquisition affects the topological organization of structural brain networks in proficient Japanese-Chinese/Chinese-Japanese speakers. Methods: A total of 33 proficient Japanese-Chinese/Chinese-Japanese speakers were recruited and divided into an early group into early and late groups according to their age of Chinese acquisition (AoCA). Sixteen of them constituted the early group, with AoCA at birth, whereas the late group consisted of 17 participants whose AoCA was after age 6. In addition to AoCA, the the age of second language acquisition (AoA-L2) was significantly younger in the early group than in the late group. All participants underwent diffusion MRI scanning. Structural brain networks were reconstructed, and graph-theoretical metrics were computed to compare topological properties between early and late groups. Results: Higher local efficiency and global clustering coefficient were found in the late group. For nodal parameters, the early group, compared to the late group, showed a higher nodal clustering coefficient in the right precuneus and higher betweenness centrality in the right Heschl’s gyrus (HG). Conclusions: Alterations in the right precuneus may be associated with earlier AoA-L2, consistent with its involvement in cognitive control processes required for managing multiple languages. Changes in the right HG could be related to earlier Chinese acquisition, particularly lexical tone learning, given the right HG’s role in pitch processing. This study showed how the linguistic environment, particularly language acquisition age, influences the topological properties of the structural connectivity, shedding light on its far-reaching effects on brain networks responsible for language processing and executive control.

## 1. Introduction

Neurolinguistics is an emerging field in which numerous researchers have investigated the role of the human brain in supporting language processing, including bilingual or multilingual abilities. Neurolinguistic studies have clarified that different language-associated experiences may induce distinct brain reorganization [1,2,3,4,5]. Among language-associated experiences, the timing of language acquisition is an essential factor (i.e., a critical or sensitive period). Put differently, the age of acquisition (AoA) in language processing is a crucial factor influencing the outcome of language learning. The effects of language acquisition age on the brain can be shown structurally and functionally. Mechelli et al. pioneered the study of the relationship between the age of second language acquisition (AoA-L2) and brain structural modulations [6]. The voxel-based morphometry technique revealed increased gray matter (GM) density in the inferior parietal cortex in early bilinguals compared to late bilinguals. This study was followed by many studies showing the AoA-L2 effects on the functional connectivity of brain networks. For example, Berken et al. showed that AoA-L2 altered resting-state functional connectivity in the language control network, presumably reflecting functional adaptation driven by second language (L2) learning [7]. Several other studies have employed a combination of morphological and functional neuroimaging, providing evidence for structure–function correspondences. For instance, Luk et al. discovered that bilingual individuals exhibited higher fractional anisotropy (FA) values, indicating greater white matter reshaping than monolingual individuals [8]. They also observed stronger functional connectivity among specific brain regions in bilinguals, including the bilateral middle temporal gyri, precuneus, right inferior parietal lobule, bilateral middle occipital gyri, and left caudate.

However, the studies above do not fully depict the relationship between AoA in language and the brain. The human brain is a complex network that is anatomically and functionally interconnected, enabling higher cognitive functions, including language processing [9]. Studies have increasingly demonstrated that language networks, instead of separate brain regions, are involved in language processing [10]. Thus, attention to neurolinguistic research has gradually shifted from analyzing the structure and function of specific brain areas to brain networks. For instance, Liu et al. explored the effects of AoA-L2 on the topological organization of the resting-state language network in highly proficient Cantonese–Mandarin speakers [11]. They found that early language learning exhibited distinct topological properties compared to late language learning. Zhao et al. also invited early and late Cantonese–Mandarin speakers to delve into their brain structural networks [12]. They discovered that early Cantonese–Mandarin bilinguals, compared with late bilinguals, exhibit significantly higher global efficiency, local efficiency, nodal efficiency, rich-club coefficient, and feeder connections, along with lower characteristic path length, indicating that an earlier AoA-L2 is associated with more efficient information exchange and integration within brain white matter structural networks. These studies suggest that language learning may be carved into the brain network architecture.

However, the two studies mentioned above on bilingual brain networks have mainly focused on similar language pairs, i.e., Cantonese–Mandarin speakers [11,12]. So, investigating linguistically less similar pairs, such as Japanese and Chinese, may provide new insights into how language AoA is related to brain organization. Chinese and Japanese are distinct in several linguistic domains, such as phonology, grammar, and syntactic structure. For example, the two languages differ in syllable inventory [13,14] and aspiration contrasts in syllable onsets [15]. In particular, one critical difference between the two languages is the use of pitch information to distinguish lexical meaning. Although both languages use pitch to distinguish lexical meaning, Mandarin Chinese relies on lexical tone much more extensively than Japanese relies on pitch accent, with approximately 71% of Chinese words requiring lexical tone to distinguish meaning, compared with about 14% of Japanese homophones distinguished by pitch accent [16,17]. Moreover, Mandarin has four contrastive lexical tones, whereas Japanese pitch accent is primarily organized through high-low pitch patterns.

This study used this less-studied language pair group to explore the AoA effects on tonal language by applying a topological analysis of diffusion MRI. Graph theory is now actively employed to explore brain networks associated with language in many respects, including as a language-deficient biomarker in disease [18] and in language learning and processing [11,19].

Accordingly, we originally intended to examine the effects of the age of Chinese acquisition (AoCA) on the topological properties of the brain in 33 highly proficient Japanese-Chinese/Chinese-Japanese bilinguals living in Japan. All the participants were classified into 16 early Chinese speakers (AoCA = at birth) and 17 late Chinese speakers (AoCA ≥ 6 years). Moreover, since Chinese was not the second language (L2) for all participants, we also collected information on their AoA-L2 for both the early- and late-Chinese-acquisition groups. We acquired their diffusion magnetic resonance imaging (dMRI) data and applied topological analysis to it.

Notably, participants in the early group had been exposed to Chinese from birth, and many of them were simultaneous Japanese-Chinese/Chinese-Japanese bilinguals. In early development, language acquisition is largely supported by auditory input and spoken interaction, making auditory–oral processing particularly relevant to the present study. Given the phonological differences between Chinese and Japanese, especially in their use of pitch, we hypothesized a priori that if AoCA influences brain organization, group differences would be most likely to involve regions associated with auditory–oral processing, particularly those implicated in lexical tone processing. In addition to language processing regions, we also considered regions involved in bilingual language control. We examined whether early L2 learning experience was associated with changes in brain networks within language-control-related regions, as previous studies have linked early bilingual experience to structural variation in these regions [20,21].

Based on these, we hypothesized that significant between-group differences would appear in areas relevant to lexical tone processing, such as the bilateral temporal gyrus (STG) [22,23,24,25]. Moreover, we expected to find differences in language-control-related areas, including the anterior cingulate cortex [1], the default mode network (DMN) [26], and the inferior frontal gyri [27].

## 2. Methods

### 2.1. Participants

We recruited 33 right-handed Japanese-Chinese/Chinese-Japanese speakers, including 16 males and 17 females aged 18–28 years (mean age ± SD = 21.48 ± 2.36 years). All were recruited for this study at various universities in Tokyo, Japan. All participants met the requirements of the Japanese school system before entering universities. All were neurologically healthy, without any health issues or contraindications to MRI. Twenty-eight participants were born in Japan, and five participants were born in China and moved to Japan in their childhood or early teen age. Written informed consent was obtained from all the participants before their participation. The protocol was approved by the ethics committee of the National Center of Neurology and Psychiatry, Japan.

Before the experiment, all participants were requested to provide detailed information about their language-learning experiences. Language history was primarily gathered through a questionnaire and interviews. The collected data included chronological age, gender, languages acquired, the respective AoA of all languages, proficiency levels in non-native languages, language exposure rate to Chinese and Japanese over the past three months, musical experience, and family language background.

According to the gathered information, our participants were categorized into two groups based on the AoCA. 6 years old is considered a critical age for L2 learning according to Qureshi (2021) and Meisel (2009) [28,29]. Moreover, by age 6, the brain reaches approximately 90% of its adult size [30,31]. Additionally, the brain has already reached 95% of its maximum volume by age 6. Therefore, we used age 6 as the cutoff to divide participants into early (N = 16) and late (N = 17) AoCA groups.

In the early group, all participants were exposed to Chinese from birth. Eleven participants acquired both Chinese and Japanese simultaneously, whereas the remaining five began acquiring Chinese from birth and Japanese later (at ages 4, 6, 12, 12, and 13 years, respectively). Among these five participants, two acquired Japanese as their L2, while the other three acquired English as their L2 at ages 6, 8, and 9 years. Excluding these three participants whose L2 was English, the remaining thirteen participants acquired English as their L3. In the late group, all participants were native Japanese speakers and acquired Chinese later in life. Two participants acquired Chinese as their L2 at ages 6 and 9 years, respectively. The remaining 15 participants acquired English (N = 14) or Korean (N = 1) as their L2 after age 8 and acquired Chinese as their L3 after age 16.

In this study, the term L2 refers to the first language acquired after the participant’s native language, irrespective of the specific language learned. Accordingly, participants who acquired two languages simultaneously from birth were considered simultaneous speakers (AoA-L2 = 0 years), whereas for sequential speakers, AoA-L2 was defined as the age at which the first additional language was introduced. Because the L2 identity varied across participants (Chinese, Japanese, English, or Korean), the analyses focused on the timing of L2 acquisition rather than on a specific language. As not all participants in the early group were simultaneous speakers, we calculated the age of the acquisition of the first non-native language (AoA-L2) in addition to AoCA. The AoA-L2 of the early group was 2.0 ± 2.9 years, and that of the late group was 12.0 ± 3.0 years.

Chinese proficiency was evaluated using two assessments: the “Hànyǔ Shuǐpíng Kǎoshì” (HSK) and the “Chinese Proficiency Test” (CPT). HSK is a standardized language proficiency test that serves as a firm reference for assessing Chinese proficiency among foreigners and Chinese ethnic minorities. In 2009, the New HSK was introduced, adopting the Common European Framework of Reference for Languages (CEFR) as the reference for assessing Chinese proficiency in China and establishing a corresponding relationship with the CEFR levels. The New HSK comprises six levels (1–6), with level 6 being the highest. It should be noted that a more recent revision, HSK 3.0, was introduced in 2021 and expanded the framework to nine levels; however, the participants in the present study were assessed using the 2009 version. CPT is primarily aimed at native Japanese speakers learning Chinese. In terms of proficiency, Quasi-Level 1 of the Chinese Proficiency Test is equivalent to level 6 of the HSK, and level 2 corresponds to a proficiency between levels 5 and 6 of the HSK. Most of our participants demonstrated high proficiency in Chinese, corresponding to the C level on the CEFR scale. Among them, 27 took the HSK test, with 17 attaining level 6 (CEFR level C2) and 10 reaching level 5 (CEFR level C1). An additional six participants completed the CPT, among whom two participants achieved Quasi-Level 1 (CEFR level C2), two reached Level 2 (CEFR level C1), one attained Level 3 (CEFR level B2), and one reached Level 4 (CEFR level B1).

We employed Language Entropy (LE) as a metric to capture both the diversity and the evenness of participants’ language usage across everyday contexts [32,33,34,35]. This measure originates from Shannon Entropy [36] and is defined by the formula $H=-\sum_{\left(i=1\right)}^{n}P_{i}\log_{2}\left(P_{i}\right)$, in which (n) denotes the total number of languages employed in a given setting, and (Pi) represents the proportion of use attributed to language (i). To obtain individual LE values, participants self-reported the number of languages they used in different contexts, along with their daily exposure rate (on a 0–1 scale) for each language over the most recent 3 months. We then computed overall LE scores from these language-use profiles using the language Entropy R package (version 1.0.1c) developed by Gullifer and Titone at McGill University, Montreal, Canada [33].

### 2.2. Image Acquisition

The participants were instructed to remain awake and lie supine on the examination bed, with their eyes closed and their heads kept still. MRI was acquired using a 3-Tesla MRI scanner (Siemens Verio-Dot, Erlangen, Germany) with a 32-channel phased-array head coil at the National Center of Neurology and Psychiatry in Japan. The DTI acquisition parameters were as follows: echo time (TE) = 83 ms; repetition time (TR) = 10,000 ms; voxel size = 2.1 × 2.1 × 3.0 mm; the number of slices = 60; slice thickness = 3 mm; field of view (FOV) = 230 mm; acceleration factor = 2; and 31 acquired volumes, including 30 diffusion-weighted directions with b = 1000 s/mm^2^ and one non-diffusion-weighted volume (b = 0 s/mm^2^).

### 2.3. Data Preprocessing

The MRI raw data were pre-processed with a Pipeline for Analyzing braiN Diffusion imAges (PANDA) (version 1.3.1, Beijing, China) [37] to obtain transformed FA images. The preprocessing of the dMRI data included the following procedures: (1) conversion of the original Digital Imaging and Communications in Medicine format into Neuroimaging Informatics Technology Initiative (NIfTI) (dcm2niigui software of MRIcron) (https://people.cas.sc.edu/rorden/mricron/dcm2nii.html) (accessed on 17 September 2021) and (2) creation of a brain mask from B0 image, eddy current correction, and the estimation of the diffusion tensor elements by using commands on FMRIB Software Library (FSL) (version 5.0.5, Oxford, UK) [38]. Subsequently, whole-brain fiber tracking and connectivity matrix construction were performed using the PANDA toolbox.

### 2.4. Network Construction

Node definition involved registering T1-weighted structural image acquired for each participant to the corresponding native FA image using an affine transformation. These transformed images were then aligned with a standard ICBM152 template in MNI space, thereby enabling the acquisition of an inverse transformation from MNI to the native space. The Anatomical Automatic Labeling (AAL) template (excluding the cerebellum) was used to divide the human brain into 90 subareas [39], which were then treated as network nodes. To achieve this, the AAL_90 template in the MNI space was aligned with the native space.

Each AAL subarea was defined as a node, and the connections between the node pairs were defined as edges. The edges were created using deterministic tractography in PANDA. The Fiber Assignment by Continuous Tracking (FACT) algorithm was used in PANDA to track the fibers throughout the brain. The tracking process followed the principal diffusion direction of each voxel, terminating when the streamline reached a voxel with FA < 0.2 or when the angle between consecutive voxels exceeded 45 degrees. The mean FA value between the two nodes was used as the edge weight. Each participant’s brain network was constructed by combining the individual parcellation map and fiber tracts, resulting in a 90 × 90 structural network matrix for each person. The data was later processed in Gretna (version 2.0.0, Beijing, China) (http://www.nitrc.org/projects/gretna/) (accessed on 3 September 2022), a toolbox for analyzing network metrics. For each participant, a threshold-based binary morphological brain network was established at sparsity levels from 0.05 to 0.5 in increments of 0.05. Using these nodes and edges, a brain network model was constructed.

### 2.5. Network Analysis

Graph theory provides a mathematical framework for modeling relational patterns in brain networks via topological metrics [40]. These metrics are broadly categorized into global and nodal parameters. Global parameters include the clustering coefficient (Cp), shortest path length (Lp), global efficiency (Eglob), local efficiency (Eloc), small-worldness (σ), normalized clustering coefficient (γ), and normalized shortest path length (λ). Briefly, Cp quantifies local clustering, Lp captures overall connectedness, Eglob reflects the efficiency of global information transfer, Eloc indexes local information exchange, and σ (the ratio of high clustering to short path length) characterizes small-world architecture. Nodal parameters include nodal efficiency, nodal clustering coefficient (NCp), and betweenness centrality (BC), which assess region-specific roles. The nodal efficiency gauges a node’s communication capacity with the rest of the network, NCp measures its local interconnectivity, and BC indicates its importance in mediating information flow. Detailed interpretations of these measures are available elsewhere [41].

### 2.6. Statistical Analysis

A chi-square test was used to detect between-group differences in gender, and an independent-samples *t*-test was applied to detect between-group differences in age and AoA. Additionally, to assess between-group differences in the topological properties of brain networks, a non-parametric permutation test was performed, with age and sex as covariates [42]. The permutation process was repeated 10,000 times to obtain an empirical distribution. The false discovery rate (FDR) approach was employed to obtain corrected multiple comparisons with a significance level of *p* < 0.05 [43]. The effect size was calculated using Cohen’s d.

## 3. Results

### 3.1. Demographic Information

The demographic information (Table 1) showed no significant difference in gender between the early and late groups, while the early group was younger than the late group (*p* = 0.018). AoCA differed between the groups as defined (*p* < 0.001, see Table 1). However, there was also a difference in AoA-L2 between the two groups (*p* < 0.001). The AoA-L2 of the early group was significantly younger than that of the late group. LE did not differ between the two groups.

### 3.2. Global Parameters and Nodal Parameters

**Figure 1:**
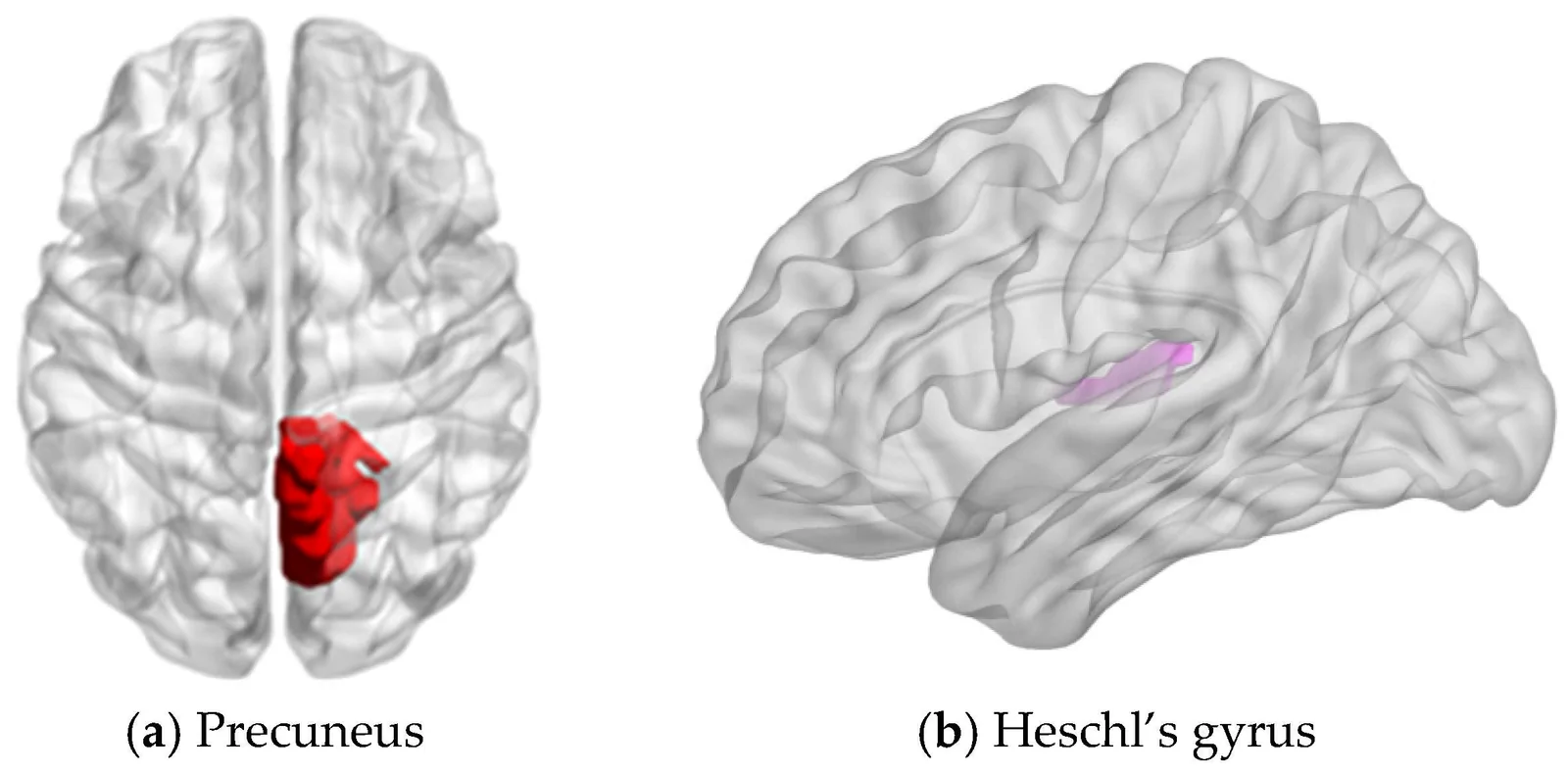
Locations of the brain areas where the early learners had higher nodal parameters than the late learners: (**a**) nodal clustering coefficient and (**b**) betweenness centrality.

In the topological analysis of dMRI-derived brain networks, the late group exhibited higher Eloc and Cp than the early learners (*p* < 0.05, FDR corrected) (Table 2). The effect sizes for Cp and Eloc were high (0.68 and 0.63, respectively). The other global parameters (Lp, Eglob, and σ) did not differ across the groups. Regarding the nodal parameters, the early group showed a higher NCp in the right precuneus (NCp of early group = 0.127, NCp of late group = 0.105, *p* < 0.001, Cohen’s *d* = 1.32) than the late group. Furthermore, the early group possessed higher BC in the right Heschl’s gyrus (HG) (BC of early group = 0.033, BC of late group = 0.031, *p* < 0.001, Cohen’s *d* = 0.52) after FDR correction (see Figure 1). No significant difference was observed in the other nodal parameters.

## 4. Discussion

Here, we compared the topological properties of diffusion tractography-based graphs between the early and late multilinguals. We found that the late Chinese speakers had significantly higher global topological parameters, Cp and Eloc, than their early counterparts. As for nodal parameters, the early Chinese speakers, who are also early L2 speakers, exhibited a higher NCp in the right precuneus and higher BC in the right HG.

### 4.1. Effects of AoA on Global Parameters

Cp serves as a comprehensive measure of interconnectivity among nodes in a network, capturing the propensity of nodes to form clusters or tightly interconnected groups. Eloc measures the degree of interconnection among the neighbors of a node, assessing how efficiently they are connected. We found that the late learners showed higher Cp and Eloc. There are many factors affecting global parameters of brain networks [44,45,46,47]. Therefore, it remains unclear whether the differences in global parameters observed here originate from the language acquisition age.

### 4.2. Effects of AoA on Nodal Parameters

#### 4.2.1. Effects of AoA-L2 on Nodal Clustering Coefficient

Nodal clustering coefficient (NCp) reflects the complexity and efficiency of local interactions in a network. The higher the clustering coefficient, the more connections the node has, and the more efficient its local interactions are. The early group showed a higher NCp in the precuneus than the late group. This finding indicates that the connections between the precuneus and its neighboring regions were more robust in the early group than in the late group. The precuneus is a crucial nexus of the DMN, which plays a role in cognitive switching [48,49]. It is believed that the precuneus has unique connections with the rest of the brain network [50]. Utevsky et al. supported the view that the precuneus interacts with the fronto-parietal network and, at the same time, with other DMN nodes, thereby distinguishing distinct cognitive states [51]. In our study, the high NCp of the precuneus may indicate that it interacts more intensively with other brain regions in early multilinguals than in late multilinguals.

It has been suggested that AoA-L2 may influence the neural mechanisms underlying cognitive control in bilinguals, particularly those involved in language switching [11]. Specifically, neuroimaging studies suggest that bilingual language processing recruits overlapping neural networks, with concurrent activation of the native language (L1) and L2 [52,53]. Our results suggested that early multilinguals may develop neural sources, including the precuneus, to facilitate language control and the interaction between L1 and L2. Previous studies have well documented the function of the precuneus in language control [54,55,56]. The simultaneous learning of two languages should induce conflicts and interferences across language representations. Switching between languages should demand attentional control to accommodate these conflicts and interference, according to the inhibitory control model [57] and bilingual interactive activation model [58]. Hence, it is necessary for early multilinguals to monitor and adjust their attention to the target language and to prevent interference from the non-target language while simultaneously outputting and comprehending the current speech flow [56]. Thus, our study suggested that earlier exposure to multiple languages likely requires greater support from the precuneus network for switching and directing attention to a particular language. The function of the right precuneus is deemed necessary to accommodate such interferences.

The increased NCp of the right precuneus in the early group is consistent with previous studies about conflicting monitoring in multilingualism. For instance, increased activation of the right precuneus was found in bilinguals during conflict-handling tasks [1]. When trilingual learners perform cognitive control tasks, the brain regions involved in resolving language conflicts and, more generally, in language processing are activated [59]. It is also argued that when words express a concept in one language, the same concept conveyed by another language is suppressed [60]. Mohades et al. consistently indicated that processing bilingual languages may affect the activation of brain regions responsible for cognitive control [59].

Previous studies have shown that early bilinguals are more strongly affected by language interference than late bilinguals because L1 and L2 are inevitably intertwined during the learning process [61,62]. It is difficult for early bilinguals to form an independent L1 lexical system at an early age. New phonological and articulatory representations are formed with the acquisition of words in L2 [63,64]. These sensorimotor representations are largely language-specific and require the establishment of distinct L2 representations, particularly when L2 learning begins later in life [63,65]. Early bilinguals may experience more frequent co-activation of L1 and L2, requiring continuous language monitoring and control during bilingual language processing. To better handle such interferences, a brain region involved in conflict monitoring would be more frequently engaged in early bilinguals than in late bilinguals. Hence, communication between the right precuneus and its surrounding nodes would be more demanding for early bilinguals than for late bilinguals because of their long-term involvement in language switching. In our study, the AoA-L2 of the early group was significantly younger than that of the late group. We propose that the immature brain of the early group in our study may develop neural mechanisms to handle language conflicts and interference, and that heightened executive control demands in early life further drive the structural reconfiguration of the precuneus.

#### 4.2.2. Effects of AoCA on Betweenness Centrality

Betweenness centrality (BC) represents the importance of a given node through which the shortest paths pass in the network. Higher BC means more shortest paths pass through the node, indicating more information exchanges through it. The early group showed higher BC in the HG than the late group, indicating that the HG is a crucial network hub that interacts with other regions to support network resilience. HG corresponds to the transverse temporal gyrus and includes the primary auditory cortex, adjoining the STG, including the sensory language center. The HG has been implicated in pitch perception and processing [66,67], auditory learning ability [68,69], and variations in accent proficiency [20]. Therefore, our interpretation of this finding is that early exposure to Chinese lexical tones may be associated with significantly higher BC in the HG observed in the early group.

Consistent with the findings of Zhao et al. [12], our results further support the view that earlier L2 acquisition is associated with more efficient brain network organization. Despite this overall consistency, our study focuses on AoA effects on Japanese-Chinese/Chinese-Japanese speakers. Unlike the Cantonese–Mandarin bidialectals investigated by Zhao et al. [12], Japanese and Chinese are two distinct languages, with one tonal and the other following a high-low pitch pattern. The differences between Chinese and Japanese are larger than those between Mandarin and Cantonese. Additionally, all participants recruited in the early group were Japanese-Chinese/Chinese-Japanese speakers who had learned Chinese from birth, whereas the early group in Zhao et al.’s research had a mean AoA for Mandarin of 3.5 years [12]. Given that the first two to three years of life represent a critical window for synaptic proliferation and myelination, this difference in early exposure timing likely leads to distinct neuroplastic outcomes. Despite these discrepancies, both studies converge on the overarching conclusion that early language acquisition significantly shapes the topological organization of brain white matter networks, underscoring the enduring influence of AoA on language-related neural structural plasticity.

The right Heschl’s gyrus has been implicated in pitch processing [70]. To satisfy the need to perceive and process a range of pitches, adequate neural resources are required. Indeed, Cai found that Chinese speakers have a larger surface volume in the HG and the planum temporale than speakers from other countries [71]. Yang et al. reported that the functional connectivity of STG between other regions in learning a tonal language is higher [72]. Crinion et al. reported higher white matter (WM) density in the right temporal lobe and the left insula in Chinese speakers than in non-Chinese speakers [73]. Native speakers of non-tonal languages who acquire speech tones presented activation in the HG [68]. Penhune et al. investigated patients with temporal lobe removals, including HG, and demonstrated the role of HG in maintaining rhythmic patterns [74]. In a word, converging evidence suggests that tonal language experiences, especially Chinese learning, may have modulated HG-connected networks associated with pitch processing.

Our study suggests that the AoCA effects, despite being confounded by AoA-L2, were most robustly represented in the right HG, a hub in a network related to pitch processing in Japanese-Chinese/Chinese-Japanese bilinguals. Additionally, the higher centrality of the right HG may contribute to more efficient network communication and integration, potentially supporting the stability of pitch-related processing. Notably, HG is a crucial hub through which sound and pitch information passes. Therefore, we speculate that the early group in our study could rely more on pitch patterns to process Chinese, resulting in a higher BC of the right HG within the local network.

## 5. Limitations

The current study has limitations. First, it is known that sample size can directly impact experimental and statistical outcomes. Although the sample size in our study is relatively small, we calculated the effect size, which suggests that the effects were robust. More participants are preferred in future studies to obtain more generalizable results. Second, Japanese and Chinese monolinguals were not included in the present study. The recruitment of Japanese monolinguals and Chinese monolinguals can be conducive to further clarifying the effects of language experience on brain networks among monolinguals, the late and early groups. Recruiting Japanese and Chinese monolinguals can help further clarify the effects of language experience on brain networks among monolinguals, particularly between the late and early groups, yielding more convincing and accurate results in the future. In addition, not all participants recruited in the study are highly proficient Chinese speakers, two of whom achieved B1 and B2 levels, indicating upper-intermediate proficiency. Fourth, we have to admit that deterministic tractography has known limitations regarding crossing fibers. Therefore, future studies using high-angular-resolution diffusion imaging techniques would be beneficial for further validating our findings. Fifth, although LE was incorporated to account for current multilingual language use, it represents only one dimension of linguistic experience. Other factors, including cumulative language exposure, educational background, and sociolinguistic environment across development, may also contribute to individual differences in brain network organization. Therefore, while our findings are consistent with an effect of AoA, the influence of these correlated experiential factors cannot be entirely ruled out. Future studies using longitudinal designs or more tightly matched multilingual cohorts are needed to further isolate the specific contribution of language acquisition timing. Finally, the two groups differed in age. The age difference between the early group (20.5 ± 1.2) and the late group (22.4 ± 2.8) might induce a confounding effect. However, multimodal evidence confirms that the two regions of interest reach topological stability by early adulthood. Lebel et al. conducted a longitudinal diffusion MRI study involving 202 healthy individuals aged 5–30 and found that most white matter fiber tracts had reached 90% maturity by late adolescence to early adulthood, while posterior and commissural fibers matured earlier, with maturation completed by approximately 11–13 years of age [75]. Both the precuneus and the HG are located in the posterior brain regions, suggesting that our participants were at an age when white matter microstructure had already stabilized. Furthermore, Chen et al., based on graph theory, further demonstrated that the most significant changes in topological indicators, such as global efficiency, local efficiency, and clustering coefficient, occurred during late childhood (9.8–12.7 years) and adolescence (12.9–17.5 years), after which the overall network tended to stabilize [76]. Among these, the precuneus, a core node of the DMN, showed a significant increase in node efficiency only during early-to-late childhood (approximately 6–13 years of age), after which it remained stable as a core hub. Regarding the HG, the classic study by Huttenlocher and Dabholkar indicated that synaptic pruning in the primary auditory cortex was largely complete by age 12 [77]. Devous et al. used Single-Photon Emission Computed Tomography to measure resting-state regional cerebral perfusion in children and adolescents aged 7–19 years and found that brain perfusion in all higher-order language areas decreased significantly with age; only the HG showed no age-related decline from age 7 to 19 [78]. The evidence above suggests that the right precuneus and right HG of the participants in our study had both entered a mature and stable phase. Therefore, the topological differences observed in these regions between the two groups of participants in this study are more likely to reflect the profound shaping of the white matter network structure in language-related brain regions by AoA.

## 6. Conclusions

In summary, the present study examined how AoA in language relates to the topology of white matter networks in Japanese-Chinese/Chinese-Japanese speakers. The early group had earlier ages of both L2 acquisition and Chinese acquisition and showed higher NCp in the right precuneus and higher BC in the right HG. Given the role of the precuneus in cognitive control, the higher NCp in it may be related to earlier AoA-L2, as acquiring and managing multiple languages from an earlier age requires sustained engagement of cognitive control processes. Similarly, earlier Chinese acquisition may be linked to structural network adaptations in the right HG. This may reflect the early demands of Chinese learning, particularly Chinese lexical tone learning, given the key role of the right HG in pitch processing. Together, these findings suggest that AoA in language is associated with region-specific features of white matter network organization, particularly in regions involved in language processing and cognitive control.

## Figures and Tables

**Table 1:** Subject Demographics.

	Early Group (N = 16)	Late Group (N = 17)	*p*-Value
Gender: male/female	6/10	10/7	0.303 ^a^
Chronological age (mean ± SD years)	20.5 ± 1.2	22.4 ± 2.8	0.018 ^b^
AoCA (mean ± SD years)	0 ± 0	17.1 ± 4.3	<0.001 ^b^
AoA-L2 (mean ± SD years)	2 ± 2.9	12 ± 2.6	<0.001 ^b^
Language Entropy	0.80 ± 0.24	0.69 ± 0.41	0.392 ^b^

^a^: chi-square test. ^b^: independent-sample *t* test.

**Table 2:** The Results of Global Parameters.

Global Parameters	M ± SD		*p*-Value	Cohen’s d
	Early Group	Late Group		
Cp	0.187 ± 0.009	0.195 ± 0.014	0.042 *	0.68
Lp	1.045 ± 0.037	1.058 ± 0.095	0.300	0.18
Eglob	0.196 ± 0.007	0.194 ± 0.016	0.349	0.16
Eloc	0.280 ± 0.012	0.288 ± 0.013	0.034 *	0.63
σ	1.408 ± 0.113	1.487 ± 0.293	0.170	0.35
γ	1.556 ± 0.136	1.677 ± 0.436	0.175	0.37
λ	0.497 ± 0.006	0.503 ± 0.026	0.227	0.31

*: a significant difference. Abbreviations: Cp, cluster coefficient; Lp, shortest path length; Eglob, global efficiency; Eloc, local efficiency; γ, normalized clustering coefficient; λ, normalized shortest path length; σ = γ/λ.

## Data Availability

The data that support the findings of this study are available from the corresponding author upon reasonable request.

## Abbreviations

The following abbreviations are used in this manuscript:

AAL	Anatomical Automatic Labelling
AoA	Age of Acquisition
AoA-L2	Age of Second Language Acquisition
AoCA	Age of Chinese Acquisition
B0	non-diffusion-weighted image (b = 0 s/mm^2^)
BC	betweenness centrality
CEFR	Common European Framework of Reference for Languages
Cp	global clustering coefficient
CPT	Chinese Proficiency Test
DMN	default-mode network
dMRI	diffusion Magnetic Resonance Imaging
DTI	Diffusion Tensor Imaging
Eglob	global efficiency
Eloc	local efficiency
FA	fractional anisotropy
FACT	Fiber Assignment by Continuous Tracking
FDR	false discovery rate
FOV	field of view
FSL	FMRIB Software Library
GM	gray matter
HG	Heschl’s gyrus
HSK	Hànyǔ Shuǐpíng Kǎoshì (汉语水平考试)
ICBM152	International Consortium for Brain Mapping 152 average template
L1	first language (native language)
L2	second language
L3	third language
LE	Language entropy
Lp	shortest path length
MNI	Montreal Neurological Institute
MRI	Magnetic Resonance Imaging
NCp	nodal clustering coefficient
NIfTI	Neuroimaging Informatics Technology Initiative
*p*	probability value (*p*-value)
PANDA	Pipeline box for Analyzing braiN Diffusion imAges
SD	standard deviation
STG	superior temporal gyrus
WM	white matter
σ	small-worldness
